# A Digital Platform (Telepalliation) for Patients in Palliative Care and Their Relatives: Protocol for a Multimethod Randomized Controlled Trial

**DOI:** 10.2196/49946

**Published:** 2024-04-02

**Authors:** Jarl Voss Andersen Sigaard, Nanna Celina Henneberg, Cathrine Skov Schacksen, Sissel Højsted Kronborg, Laura Petrini, Kristian Kidholm, Una Rósa Birgisdóttir, Helle Spindler, Birthe Dinesen

**Affiliations:** 1 Hospital of Southwest Jutland University Hospital of Southern Denmark Esbjerg Denmark; 2 Laboratory for Welfare Technology—Digital Health & Rehabilitation ExerciseTech, Department of Health Science and Technology Aalborg University Aalborg Denmark; 3 Center for Neuroplasticity and Pain Department of Health Science and Technology Aalborg University Aalborg Denmark; 4 Centre for Innovative Medical Technology Odense University Hospital University of Southern Denmark Odense Denmark; 5 Department of Psychology and Behavioural Scienses Aarhus University Aarhus Denmark

**Keywords:** palliative care, digital health, quality of life, telehealth, interdisciplinary research, randomized controlled trial

## Abstract

**Background:**

The World Health Organization defines end-of-life palliative care as “prevention and relief of suffering, by means of early identification and impeccable assessment and treatment of pain and other problems, physical, psychosocial and spiritual.” Over 20 million people worldwide are in need of palliative care. In Denmark, palliative care is given at a general and a specialist level. The general level comprises health care professionals (HCPs) who do not perform palliative care full-time. The specialist level comprises specialized palliative care (SPC), where HCPs perform palliative care full-time. In total, 20%-30% of patients who need palliative care are referred to SPC. Challenges with SPC include a short time span from referral to end of life, patients who are very ill and may therefore find it hard to travel to an outpatient clinic, and the SPC unit having a relatively small staff. The need for SPC is expected to rise, as the number of patients dying from terminal diseases is increasing. Telehealth has been successfully implemented in different home care settings, including palliative care.

**Objective:**

The aim of the study is to present the research design of the clinical testing of a telepalliation program by the use of a digital platform for patients in palliative care and their relatives.

**Methods:**

The telepalliation program will be conducted as a multimethod randomized controlled trial. The intervention group will follow the telepalliation program, while the control group will follow the traditional standard of care program for palliative care. The primary outcome of the study is increased quality of life. Secondary outcomes include enhanced sense of security; reduced experience of pain; satisfactory experiences of patients and relatives with the TelePal platform and degree of satisfaction in being a part of the program; experiences with the use of the TelePal platform on the part of HCPs and the professionals’ experiences of being a part of the program; the use of a cross-sector communication platform and the telepalliation program by patients, relatives, and HCPs; and the projected lower cost of health care services. These outcomes will be assessed using questionnaires, data generated by digital technologies, and semistructured interviews.

**Results:**

The collection of data began in May 2021 and will be completed in August 2024. The results of the study will be published in peer-reviewed journals and presented at international conferences. Results from the telepalliation program are expected to be published by fall 2024.

**Conclusions:**

The expected outcomes of the study are increased quality of life and increased sense of security. We also expect that the study will have a clinical impact on future telepalliation for those patients who are referred to a palliative team.

**Trial Registration:**

ClinicalTrials.gov NCT04995848; https://clinicaltrials.gov/study/NCT04995848

**International Registered Report Identifier (IRRID):**

DERR1-10.2196/49946

## Introduction

The World Health Organization defines end-of-life palliative care as “prevention and relief of suffering, by means of early identification and impeccable assessment and treatment of pain and other problems, physical, psychosocial and spiritual” [1]. The aim of palliative care is to provide relief from pain and other distressing symptoms offered by health care professionals (HCPs) at a general or specialist level, with a focus on addressing the needs of patients and their families [1,2]. More than 20 million people worldwide are estimated to be in need of end-of-life palliative care, equivalent to 377 adults in a population of 100,000, the majority of these being adults aged 60 years and older [2].

In Denmark, as in most other Western European countries, palliative care is provided at 2 different levels, a general and a specialist. The general level comprises HCPs, such as general practitioners (GPs) and primary care nurses, who do not perform palliative care on a full-time basis. The specialist level comprises specialized palliative care (SPC), where HCPs perform palliative care on a full-time basis at a specialist level [3]. It is expected that the basic level palliative care can address around 70%-80% of the patients in need of palliative care. The remaining 20%-30% of patients, having more complex problems, need SPC. SPC consists of specialized palliative care teams (SPCTs) placed at large hospitals serving a wider geographical area with home visits and cross-sector communication with patients, relatives, and other HCPs capable of caring for the patients.

Among patients receiving SPC, more than 90% are diagnosed with cancer [4], with the remaining cases divided among patients diagnosed with terminal cardiac insufficiency, terminal chronic obstructive pulmonary disease, and terminal neurological disorders [2,4].

There are several challenges associated with SPC, such as the very short time span from referral to the end of the patient’s life [4] and the fact that patients are very ill and may therefore find it hard to travel to an outpatient clinic and that SPC unit often has a relatively small staff [2,5-7]. The need for SPC is expected to rise in the future, as the number of patients dying from life-threatening diseases, including cancer, is increasing [5,8]. As an increasing number of these patients prefer to be cared for at home, the demand for SPC is expected to increase [5,8].

The use of telehealth has been introduced in different settings and at different levels of home care and has also been used with success in palliative care [8,9]. Demand for telehealth solutions in palliative care has also increased due to the COVID-19 pandemic [10,11]. The use of information and communication technologies within palliative care can be defined as “telepalliation.”

The overall purpose of the telepalliation study is to test, implement, analyze, and evaluate a telepalliation program for patients receiving palliative care. To accomplish this goal, we combine clinical, psychosocial, interorganizational, and health economic approaches. The telepalliation study will be implemented and evaluated in a randomized controlled trial (RCT). The aim of this paper is to describe the research design of the telepalliation study, the data collection methods used, and the outcome measures.

## Methods

### Research Design

The telepalliation program will be conducted as a multimethod RCT using qualitative and quantitative data collection techniques [12,13]. The intervention group will take part in the telepalliation program, while the control group will follow a traditional palliation program at Southwest Jutland Hospital, Esbjerg, Denmark. Enrollment of patients began in May 2021, and the RCT will end in August 2024. The study will be reported following the SQUIRE (Standards for Quality Improvement Reporting Excellence) guidelines [14].

The purpose of the telepalliation study is to test, implement, and evaluate a telepalliation program for patients receiving SPC at the Hospital of Southwest Jutland, Esbjerg, Denmark. To accomplish this goal, we combine clinical, psychosocial, interorganizational, and health economic approaches. This will be done by collecting data on symptom scores over time, patients scoring their feeling of security over time, and interviews with patients, their families, HCPs of the specialist team, and primary HCPs. Interviews will focus on the use of the TelePal platform but also on the interorganizational perspective of the communication between primary and secondary health systems. Evaluation of overall economic implications will be done separately at the end of the study. The telepalliation study will be implemented and evaluated in an RCT.

### The TelePal Platform

The telepalliation program and platform have been developed through a participatory design process [15], involving patients who have received SPC and their relatives, HCPs working in palliative care, district nurses, GPs, and an interdisciplinary research team.

The study uses a TelePal platform, a communication platform that is shared among the patient, his or her relatives, and related HCPs. The platform can be used to communicate between patients, their families, and HCPs in primary and secondary health care. The patients must give their written consent as to whom they want to share their data. All information will be available and presented in the same way to all participating parties. The HCPs and researchers have extra tools with which to administer care, gain an overview of the patient’s condition, and monitor multiple patients at the same time. The digital platform is not an electronic patient record but a platform for coordination and communication across sectors between patients, relatives, and HCPs.

**Figure 1:**
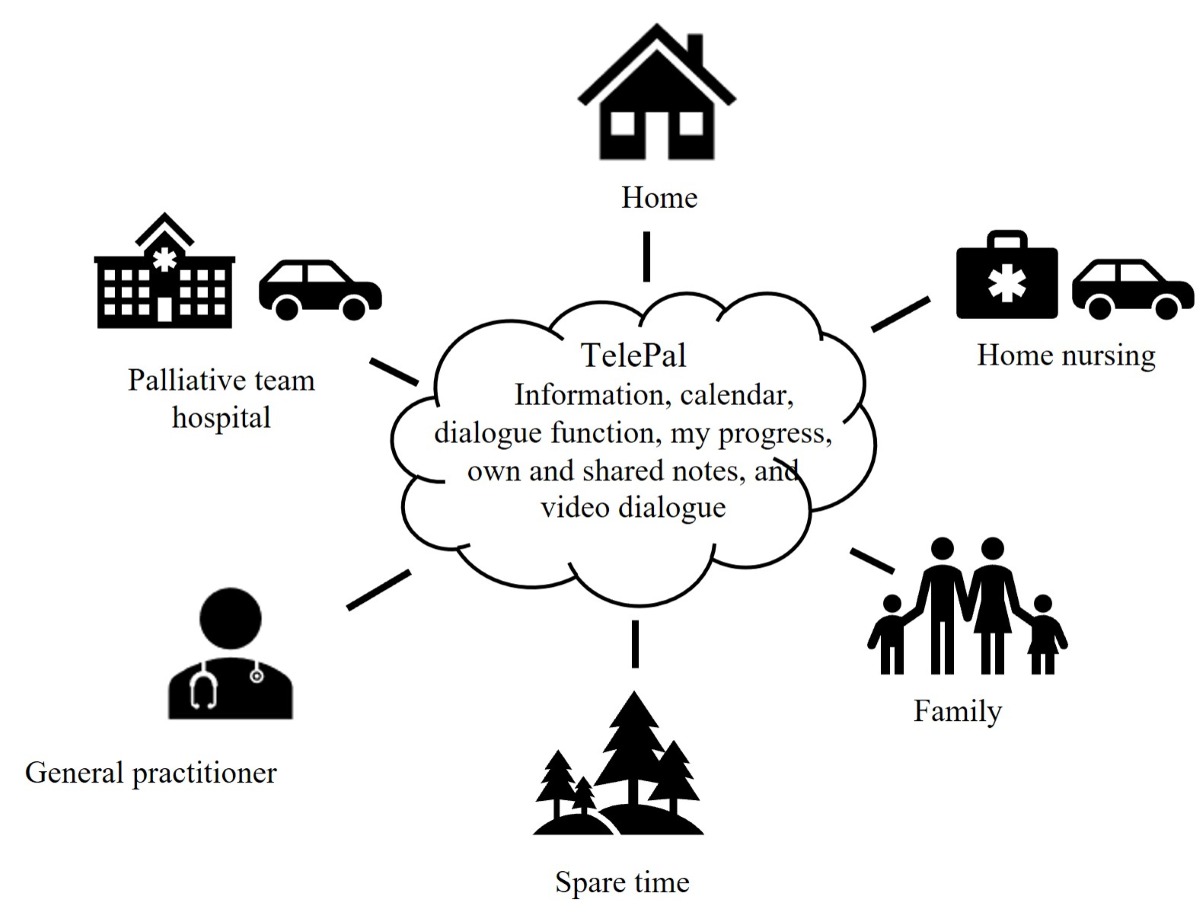
Overview of the TelePal platform.

All the measured values are stored on a secure database at Aalborg University (AAU). The TelePal web portal (Figure 1) is a web-based platform that encompasses multiple features that have been selected and developed through user-driven innovation [16]. Facilities and entered data (symptom scores, etc) of the specific patient are available to the persons, to whom the patient allows access to the platform. The features are described in Textbox 1.

**Features of the TelePal web portal.**

**My page/front page (*Min side* in Danish); used to give the patient an overall overview of the different features)**

- New messages
- Video consultations (the patient can have video conversations with the palliative team, district nurses, and GPs via TelePal. However, only the HCPs can book meetings with patients and their relatives)
- Questionnaires for patients to fill out

**My treatment (*Mit forløb* in Danish)**

- Dialogue function (here, the patients can send and reply to messages to the palliative team and the district nurses; if the patient so desires, an SMS function can be used to inform the patient about new messages on TelePal)
- Calendar (gives the patient the opportunity to register coming appointments with the palliative team or any other activity for an overview)
- Joint notes (notes related to the patient’s treatment are written here by either the palliative team, the home nurse, or GPs)
- Own notes (these are the patient’s own notes, thoughts, questions, and reflections, if they want to use this function)

**My status (this site provides the patient with an overview of the results from all the questionnaires they have filled out)**

- The data from the questionnaires are visible to the patient’s relatives and HCPs, if the patient has allowed them to see and follow their data on TelePal.
- Patients may decide themselves whether they want to allow up to 2 relatives to have access to their TelePal platform. It is also the patient who defines what relatives should have access.

### Data and Network Security

The TelePal platform is web-based. It is possible to access the platform from any internet-enabled device, for example, a PC or Mac computer or a tablet, via any modern browser. The most optimal experience and resolution will appear when using an iPad or tablet. All communication and data are secured based with SSL and a 2-factor log-in. The portal will be kept at AAU on servers provided by AAU IT services.

### Eligibility Criteria

All participants will be recruited by 2 project nurses at the palliative team at Southwest Jutland Hospital, Esbjerg, Denmark. Recruitment will take place by personal contact and by a recruitment letter. Enrollment will proceed if the participant fits the inclusion and exclusion criteria. Inclusion criteria for participants are that they must be patients under SPC at the Hospital of Southwest Jutland; be aged 18 years or older; live in Esbjerg, Varde, Billund, Fanø, or Vejen municipalities; have cancer, heart failure, chronic obstructive pulmonary disease, or motor neuron disease; and have basic computer skills or have a relative who is able to help them. The exclusion criteria are delirium at enrollment based on the Confusion Assessment Method score; an active psychiatric history other than depression or anxiety related to the main diagnosis, which refers to the reason for palliative care; and a lack of cooperation. Enrollment will take place at the patient’s home, where a doctor and a nurse from the palliative team will take the inclusion and exclusion criteria into consideration. Patients who are moribund are not assessed for participation in the study. The patient will be given complete information about the project, and the palliative team will obtain informed consent. The project nurse will then proceed to explain to the patient how to use the equipment and the TelePal portal. If the patient meets the inclusion criteria and agrees to be included, randomization to either the intervention group or standard care will be made.

### Details on the RCT

The randomization is performed randomly by a digital tool. The randomization was designed as a block with equal numbers for the intervention and control groups. Only 1 person was blinded. The intervention group participated in the telepalliation program, and the control group received standard SPC [1,3] that was individualized for each patient.

### Sample Size

The sample size of the telepalliation study was determined to be 91 patients in both the intervention and the control groups when calculating values based on Ramsenthaler et al [17]. This was done using a 95% CI, an 80% power, an SD of 20.5, a mean quality-of-life score of 59.5 determined by the European Organisation for Research and Treatment of Cancer, and a mean quality-of-life score of 68.43 determined by the European Organisation for Research and Treatment of Cancer when operating with a 15% change in quality of life (QoL).

### Theoretical Framework

The theoretical framework is applied as lenses in qualitative analysis.

#### Psychosocial

In recent years, increasing attention has been paid to the importance of psychosocial factors in palliative care and how these factors can play a crucial role for good end-of-life care in terms of alleviating pain and other distressing symptoms [18]. The World Health Organization [1,19] stated that control of pain, psychological disorders like anxiety and depression, and social and spiritual problems is paramount in palliative care.

Research shows that an intervention focused on the assessment of pain and psychosocial symptoms, establishment of goals of care, assistance with decision-making regarding treatment, and individualized coordination of care can all significantly improve patient QoL. Patients reported less depression and physical symptom burden and lived an average of 2.7 months longer than the usual care group, despite receiving less aggressive care [20].

#### Interorganizational Theory

Collaboration across sectors in the telepalliation program will be studied through the lenses of interorganizational theory by Alter and Hage [21]. The network approach facilitates an exploration of the interplay, communication, collaboration, and dynamics between the HCPs, for example, palliative team, district nurses, and GPs across borders and sectors when developing and implementing the telepalliation program. Alter and Hage [21] define a network as follows: “Networks constitute the basic social form that permits inter-organizational interactions of exchange, converted action, and joint production. Networks are unbounded or bounded clusters of organizations that, by definition, are non-hierarchical collectives of legally separate units.”

### Ethical Considerations

This study was approved by the ethical committee in Northern Jutland (N-202000094) and registered at ClinicalTrials.gov (NCT04995848). This study will be conducted according to the Declaration of Helsinki. Patients in palliative care and their relatives are a vulnerable group and will be informed thoroughly about the purpose of the study. Accordingly, it is specified that the patient can withdraw their consent at any given time in the study without having any consequences for their treatment. Individuals will be considered as participants in the trial, whereas their relatives will not be considered as participants. However, they are invited to voluntarily participate in the use of the digital platform TelePal. To clarify and ensure that all relatives are fully informed about the study and feel safe about their participation, a written letter of information was sent to all relatives. Patients are enrolled in the study for a maximum of 6 months or until (1) they stop being followed by the palliative team due to lack of symptoms or (2) they are diagnosed with delirium based upon the Confusion Assessment Method score [22,23]. Patients who develop cognitive impairment will be dismissed from the study, and patients who develop delirium will also be dismissed, and data collection will end immediately.

### Baseline Data

Baseline data on demographics and diagnosis will be collected for both the intervention and the control groups. Questionnaires regarding QoL, sense of security, pain assessment, and quality-adjusted life years (QALYs) will be combined in questionnaire packages matching the time of measurement illustrated in Table 1. The intervention and the control group will be asked to fill in identical questionnaire packages at the same point in time. All years will be answered by both the intervention and the control group, thereby meaning that both groups obtain identical questions and the same number of questionnaires. All questionnaires will be filled out on TelePal. However, the control group can choose to complete the questionnaires on paper if they desire.

**Table 1:** Primary and secondary outcome measures.

Outcomes		Time of measurement	Group	
			Intervention	Control
**Primary**				
	Quality of life	Once weekly	✓	✓
**Secondary**				
	Changes in medicine	Weekly for 6 months	✓	✓
	Pain assessment	Weekly	✓	✓
	Feeling of security	Twice a week	✓	✓
	Patients’ experiences	Interviews will be conducted after 4 weeks	✓	
	Relatives’ experiences	Interviews will be conducted after 4 weeks and after 3 months	✓	
	Health care professionals’ experiences	Interviews will be conducted at 6 and 12 months		
	Use of the TelePal platform and telepalliation program	Analysis at the end of the project	✓	✓
	Cost of health care services	Week 1 and week 4, and analysis at the end of the project	✓	✓
	Quality of life associated with cost of health care services	Week 1 and week 4, and analysis at the end of the project	✓	✓

### Outcome Measures

#### Overview

Primary and secondary outcome measures will be collected as shown in Table 1. The data collection process is described in the following sections.

#### Quality of Life

The EORTC QLQ-C15-PAL (European Organisation for Research and Treatment of Cancer Quality of Life Questionnaire Core 15 Palliative Care) [24] questionnaire will be used to measure QoL as the primary outcome. The EORTC QLQ-C15-PAL was developed by the European Organization for Research and Treatment of Cancer to measure the QoL for patients in palliative care. It is a briefer version of the EORTC QLQ-C30-PAL I, as this patient group is often not able to complete extensive self-report measures. It consists of 15 items, of which, all but one (global QoL) is self-rated on a 4-point Likert scale (1=not at all to 4=very much), whereas global QoL is rated on a 7-point Likert scale (1=very poor to 7=excellent). In this study, we did not use the global QoL item. Items are used to calculate scores on 2 multi-item functional scales (emotional and physical), 2 multi-item symptom scales (fatigue and pain), and finally, they contribute to 5 single-item symptom scales (nausea or vomiting, lack of appetite, shortness of breath, constipation, and sleeping difficulties). The EORTC QLQ-C15-PAL is considered to have good content validity [24], and a recent study comparing measures of QoL in palliative care found acceptable internal consistency and test-retest reliability as well as sensitivity of the EORTC QLQ-C15-PAL, while overall results indicated that no other QoL measure is superior to use in this patient group [25].

In addition, the EQ-5D health questionnaire was used to determine QALY to support our analysis on the cost of health care services, as no other QoL measure is validated for this purpose. Hence, we incorporated EQ-5D, the EQ Visual Analog Scale from the EQ-5D, which records the patient’s self-rated health on a vertical visual analog scale using the end points, the best health you can imagine and the worst health you can imagine. This scale can be used as a quantitative measure of health outcomes that reflect the patient’s own judgment [26]. Studies have shown good construct validity and responsiveness in palliative care [27], and a recent systematic review finds that the EQ-5D has excellent psychometric properties across a broad range of populations [28].

#### Changes in Medicine

Information on medicine for both groups will be collected at enrollment and every week from the electronic patient record during a 6-month period. Changes in medicine over time will be analyzed.

#### Pain Assessment

In line with the recommendations for supplementing the EORTC QLQ-C15-PAL with focused measures, if more information on a specific content area is warranted [29], and in accordance with the Expert Working Group of the European Association for Palliative Care [30], we incorporated the Brief Pain Inventory Short Form, a pain measurement tool [31]. The Brief Pain Inventory rapidly assesses the severity of pain and its impact on the patient’s functioning. In this study, we incorporated the 11-point numeric rating scale (0-10) for pain intensity as well as 7 items focusing on the impact of pain on functioning in relation to activity, mood, ability to walk, work, relation to other people, sleep, and general joy of life rated on an 11-point numeric rating scale (0=no impact to 10=full impact). The internal consistency of the Brief Pain Inventory (Cronbach α) ranges from .77 to .91, and more specifically, for the pain intensity scale, Cronbach α ranges from .78 to .96 [32].

#### Sense of Security

To evaluate the patient’s sense of security using the TelePal platform, we developed a single item (“Overall, how would you rate your sense of security over the past 24 hours?”), rated on a 5-point scale (1=very unsafe to 5=very safe), to be used in this study.

#### Use of TelePal Platform and Experiences Using the TelePal Platform

Qualitative exploration of the patients’, their relatives’, and the HCPs’ experiences using the TelePal platform will be collected during semistructured interviews inspired by Brinkmann and Kvale [33]. Patients will be selected randomly to participate in interviews. Interviews with patients and relatives will be conducted after 4, 8, and 12 weeks. Interviews with HCPs will be conducted after 6 and 12 months. All interviews with patients, relatives, and HCPs will be conducted until data saturation has been reached. To analyze which parts of the TelePal platform are being used and for how long, time log files for log-in and log-out of patients, relatives, and HCPs will be analyzed at the end of the project. Consent for the extraction of log files from the database will be received from patients, relatives, and HCPs.

#### Evaluation of Cost of Health Care Services

The data used to determine QALYs will come from the EQ Visual Analog Scale of the EQ-5D questionnaire [26] (see description in the Quality of Life section). Patients will be asked to answer the EQ-5D health questionnaire twice, first in week 1 of the study and then in week 4, in order to determine QALYs [26]. The questionnaire will be completed by the intervention group on TelePal, and the control group will complete the questionnaire on TelePal or on paper. Furthermore, a cost-effectiveness analysis will be conducted by calculating the differences in clinical efficacy and differences in average cost per patient [34,35]. The cost parameters are numbers of phone or video calls from the palliative team, equipment used, driving distance, personal use in palliative care, visits from GPs, outpatient clinic visits, numbers of hospitalizations, readmissions, length of stay, and visits from the palliative team.

### Adverse Events and Dropouts

All adverse events, deaths, dropouts, or withdrawals from the study will be recorded and documented. If the patients no longer want to participate in the study, they can withdraw their consent at any time without having to give a reason. After withdrawing they will be given standard care treatment. The patient’s reason for withdrawal, if any is given, will be documented. In this case, the project team will collect the equipment upon request. Patients who do not participate actively in the intervention will still be included in the study and analyzed according to the intention-to-treat approach. These patients will be allowed to use the project equipment as long as they want. Technical problems with the equipment will be recorded and documented.

### Statistical Analysis

The applied statistical methods will be used to investigate differences between patients in the intervention group and the control group. Statistical analyses will be used to explore the normal distribution of the sample, the SD, the hypothesis tests, and the *P* values. Nevertheless, a 2-tailed *t* test between the intervention and control groups will be performed in order to compare baseline data between the 2 groups, and a 2-way ANOVA with repeated measures will also be performed to compare the 2 groups in relation to their outcomes. All the statistical analyses will be performed using SPSS Statistics (version 25; IBM Corp). Data will be analyzed for gender differences in both primary and secondary outcomes. If there is any missing data, it will be analyzed based on the data received from the questionnaires. If there is too much data missing, a sensitivity test will be performed based on the demographic data.

### Qualitative Analysis

All interviews will be transcribed into Word (Microsoft Corp) files. The data will be coded in NVivo (version 12.0; QSR International) inspired by methods developed by Brinkmann and Kvale [33]. Two researchers (JVAS and BD) will conduct the interviews and analysis of the data. The data will be presented in themes, findings, and with citations.

## Results

Results from the RCT will be analyzed in spring 2024, published in peer-reviewed journals in the fields of palliative care and telepalliation, and presented at relevant international conferences by fall 2024.

## Discussion

### Principal Findings

The aim of the telepalliation study is to test, implement, and evaluate the telepalliation program for patients receiving palliative care. The study is carried out using combined clinical, psychosocial, interorganizational, and health economic approaches.

A scoping review by Steindal et al [36] has shown that the use of telehealth in palliative homecare improves access to HCPs at home and enhances patients’ sense of security and safety. The review indicated that there are contradicting results as to whether the use of telehealth will improve burdensome symptoms and QoL [36]. A study by Caraceni et al [11] has shown that telemedicine facilitates patient-clinician interaction, but the investigation of clinical impact should be better documented.

The TelePal platform and program have been developed in a participatory design process, and an important part of the study is to explore how patients in palliative care and their relatives experience the TelePal program and use the digital platform. Important questions to explore are whether the patients have the resources to use the technology, and if so, what functions are they and their relatives using and what value do they attribute to these functions. Another important question revolves around the ethical issues of using a digital platform in the collaboration between patients in palliative care and HCPs. Steindal et al [36] stated that there is a need for increased knowledge about these issues. We hope that the platform can help increase the feeling of security for the patients and relatives and ease communication, collaboration, and coordination among the HCPs across sectors.

In palliative settings, video has been used for conferences between patient’s homes and between rural health professionals and specialist centers to support patients and their relatives at home [8]. The use of video can increase the collaboration between SPCTs and GPs [6] and fit the practice of home-based palliative care, thus resulting in a more empathetic patient-professional relationship [37]. In the TelePal study, we will explore how the platform will affect the patient care process and interorganizational collaboration among the HCPs across sectors. We have not identified other studies using a telepalliation platform and a program.

What are the health economy costs of running a telepalliation program? Caraceni et al [11] highlighted the lack of cost-effectiveness studies within telepalliation. In the TelePal study, we will conduct a cost-effectiveness evaluation to show that it will be cost-effective, reduce the cost for transportation, and reduce patients’ travel to the hospitals.

### Limitations

A limitation of this study is that it is a 1-center study and therefore reflects the findings of only a single SPCT. The fact that it has been conducted only in Denmark means that it reflects findings within a Danish context. These findings may not necessarily be applicable to other settings and in different contexts.

### Conclusions

The expected outcomes are increased QoL and increased sense of security for patients in the intervention group. We expect that the study will have a clinical impact on future telepalliation for patients who are referred to a palliative team.

## Abbreviations

AAU: Aalborg University
EORTC QLQ-C15-PAL: European Organisation for Research and Treatment of Cancer Quality of Life Questionnaire Core 15 Palliative Care
GP: general practitioner
HCP: health care professional
QALY: quality-adjusted life year
QoL: quality of life
RCT: randomized controlled trial
SPC: specialized palliative care
SPCT: specialized palliative care team
SQUIRE: Standards for Quality Improvement Reporting Excellence
